# *Vanda roxburghii* chloroform extract as a potential source of polyphenols with antioxidant and cholinesterase inhibitory activities: identification of a strong phenolic antioxidant

**DOI:** 10.1186/s12906-015-0728-y

**Published:** 2015-06-23

**Authors:** Md. Nasim Uddin, Rejina Afrin, Md. Josim Uddin, Md. Jalal Uddin, A. H. M. K. Alam, Aziz Abdur Rahman, Golam Sadik

**Affiliations:** Department of Pharmacy, University of Rajshahi, Rajshahi, 6205 Bangladesh

**Keywords:** Alzheimer’s disease, *Vanda roxburghii*, Cholinesterase inhibition, Antioxidant activity, Gigantol

## Abstract

**Background:**

Alzheimer’s disease (AD) is a progressively developing neurodegenerative disorder of the brain in the elderly people. *Vanda roxburghii* Rbr. root has been used traditionally in Bangladesh as tonic to brain and in the treatment of nervous system disorders including AD. Therefore, we aimed to investigate the cholinesterase inhibitory activities and antioxidant properties of the extracts from *V. roxburghii*.

**Methods:**

The crude methanol extract from the roots of plant was sequentially fractionated with petroleum ether, chloroform, ethylacetate and water to yield their corresponding extracts. The extracts were assessed for acetylcholinesterase and butyrylcholinesterase inhibitory activity by modified Ellman method and antioxidant property by several assays including ferric reducing antioxidant power, scavenging of 1,1-diphenyl-2-picrylhydrazyl (DPPH) free radical and hydroxyl radical, and inhibition of lipid peroxidation. Endogenous substances in the extracts were analyzed by the standard phytochemical methods and active compound was isolated by the chromatographic methods.

**Results:**

Chloroform extract was shown to demonstrate strong ferric-reducing antioxidant power and scavenging activity against DPPH and hydroxyl free radicals when compared with the other extracts and the reference standard catechin. The antioxidant effect was further verified by inhibition of lipid peroxidation in rat brain homogenates. Likewise, the chloroform extract exhibited the highest inhibition against both the acetylcholinesterase and butyrylcholinesterase enzymes with IC_50_ values of 221.13 and 82.51 μg/ml, respectively. Phytochemical screening revealed a large amount of phenolics and flavonoids in the chloroform extract. Bioactivity guided separation techniques led to the isolation of a strong antioxidant from the chloroform extract and its structure was determined as gigantol on the basis of spectral studies.

**Conclusion:**

These results suggest that the chloroform extract of *V. roxburghii,* possibly due to its phenolic compounds, exert potential antioxidant and cholinesterase inhibitory activities, which may be useful in the treatment of AD.

## Background

Alzheimer’s disease (AD) is the most prevalent neurodegenerative disorder in the elderly people with symptoms of memory loss, cognition and behavioral abnormalities. The major neuropathological characteristics observed in AD are senile plaque consisting of Abeta protein, and neurofibrillary tangles of microtubule associated protein tau, and loss of cholinergic neurons [1–4]. Currently there is no definitive treatment or cure for this disease. Extensive biochemical studies with AD revealed cholinergic dysfunction and oxidative stress as the major contributing factors in the pathogenesis [4, 5]. It is well established that the cholinergic system, which is responsible for the storage and retrieval of items in memory, is impaired in AD [6, 7]. The reduced cholinergic transmission and the associated decrease of acetylcholine (ACh) have been shown to be critical in the development of dementia [8]. Therefore, inhibition of acetylcholinesterase (AChE) as well as butyrylcholinesterase (BChE), which are involved in the breakdown of acetylcholine, has been the main strategy followed for the treatment of AD [9, 10]. Until now, only three cholinesterase inhibitors (ChEI) such as donepezil, galantamine and rivastigmine has been approved by the US Food and Drug Administration (FDA) to treat AD [11, 12]. These drugs are effective only in mild to moderate AD and do not reverse the disease progression. Moreover, they are associated with multiple side effects [13]. Therefore, it has become a necessity to develop the new ChE inhibitors that are pharmacologically safe, cost effective and immediately available with minimal side effects.

Oxidative stress (OS) has been shown to be another important factor in the pathological cascade of AD. Oxidative stress markers including lipid peroxidation, modification of DNA, protein oxidation and reactive oxygen species (ROS) formation has been documented in the brains of AD [14–16]. Numerous studies suggested that oxidative stress and amyloid A-beta protein are linked each other because amyloid A-beta induces oxidative stress *in vivo* and *in vitro* [14, 15], and oxidative stress increases the production of amyloid A-beta [16, 17]. Oxidative stress increases is believed to be an early event in AD pathology [18, 19] as it contributes to membrane damage, cytoskeleton alterations and cell death [20]. Therefore, antioxidant therapies have been suggested as a potential means to reduce reactive oxygen species (ROS)-based damage in AD [21, 22]. Plants rich in polyphenol and flavonoids are important sources of antioxidants and have shown effective in oxidative damage of tissue [23].

*Vanda roxburghii,* belonging to the family Orchidaceae, is an epiphyte and widely distributed throughout Bangladesh. Medicinal uses of this plant have been described well in both Unani and Ayurveic systems of medicine [24–26]. In Yunani medicine, the root is used as tonic to the liver and brain and also indicated in the treatment of bronchitis, piles, lumbago, toothache, boils of the scalp and fractures [24]. The plant is traditionally used by the local people of Bangladesh to treat diseases of nervous system including AD [25]. According to ethnomedical uses, the root of plant is reputed to have activity against inflammations, bronchitis, rheumatic pain, disease of abdomen, tremor and infectious diseases including bacterial infections and tuberculosis. The leaves are pounded and the paste is applied to the body to bring down fever; their juice is dropped in the ear for the treatment of otitis and other inflammatory conditions [26]. A comprehensive review of literature revealed that the plants of this family has some promising biological activities like antiinflammatory, antiarthritic, antimicrobial, anticancer, antioxidant, antinociceptive, and wound healing properties [27–33]. Previously we reported the antibacterial activity of the methanol extract of *V. roxburghii* [33]. Given the ethnomedicinal uses of this plant in the nervous system disorders, we have undertaken to evaluate the cholinesterase inhibitory activity and antioxidant property of the extracts from *V. roxburghii*.

## Methods

### Chemicals

DPPH (1, 1′-diphenyl-2-picrylhydrazyl), aluminum chloride, ammonium molybdate, potassium ferricyanide, ascorbic acid, Folin-ciocalteu reagent, Tris–HCl and triton X-100 were obtained from Sigma-Aldrich, India. Gallic acid was obtained from Wako Pure Chemical Company Ltd., Japan. 2-deoxy-D-ribose, 2-thiobarbituric acid (TBA), catechin, quercetin, 5,5′-dithio-bis-(2-nitro) benzoic acid (DTNB), acetylthiocholine iodide, S-butyrylthiocholine, galantamine, and donepezil were obtained from Sigma-Aldrich, Japan. Unless otherwise specified, all other chemicals were of analytical grade.

### Plant material

The roots of *V. roxburghii* were collected from Rajshahi University campus, Rajshahi, Bangladesh, and identified by an expert taxonomist. A voucher specimen (collection no. 92) was submitted to the herbarium of the Department of Botany, Rajshahi University.

### Extraction

Fresh roots of *V. roxburghii* were thoroughly washed, crushed into small pieces, shade-dried and ground into coarse powder by using grinding mill. Dried powder (500 g) was extracted with methanol for 7 days at room temperature with occasional shaking and stirring. The extract was then filtered and concentrated with a rotary evaporator under reduced pressure at 50 °C temperature to afford the crude methanol extract (12.5 g). An aliquot (10 g) of the concentrated methanolic extract was partitioned by the method as described earlier [34] and the resultant partitionates that is petroleum ether (PEF, 2.05 g), chloroform (CLF, 3.68 g), ethylacetate (EAF, 1.67 g) and aqueous (AQF, 2.6 g) extracts were obtained and used for the experiment purpose.

### Phytochemical screening of the plant extract

Preliminary qualitative analysis of the extracts were carried out to determine the presence of various phytochemicals which include tannins, phenolics and flavonoids, alkaloids, saponins, and steroids in accordance with the methods as described [34].

### Determination of total phenolic content (TPC)

The TPC of the different extracts of *V. roxburghii* was determined using the Folin-Ciocalteu reagent [35]. 0.5 ml of plant extract or standard solution at different concentrations was added to 2.5 ml of Folin – ciocalteu (diluted 10 times with water) reagent and 2.5 ml of sodium carbonate (7.5 %) solution. The reaction mixture was incubated for 20 min at 25 °C to complete the reaction and the absorbance of the mixture was measured at 760 nm. Gallic acid was used as standard and the results were expressed as mg of gallic acid equivalent (GAE)/g of dried extract.

### Determination of total flavonoid content (TFC)

The TFC of the different extracts of *V. roxburghii* was determined by aluminum chloride colorimetric method using catechin as a standard [36]. The plant extract (1.0 ml) was added to 3.0 ml of methanol, 0.2 ml of 10 % AlCl_3_, 0.2 ml of 1 M potassium acetate and 5.6 ml of distilled water. The reaction mixture was then incubated at room temperature for 30 min to complete the reaction. The absorbance of the mixture was measured at 420 nm. Catechin was used as standard and the results were expressed as mg of catechin equivalent (CE)/g of dried extract.

### Determination of ferric reducing antioxidant power (FRAP)

The FRAP of different extracts of *V. roxburghii* was evaluated by the method of Oyaizu [37]. Plant extract or standard solutions at different concentration (1 ml) were mixed with 2.5 ml each of potassium phosphate buffer (0.2 M) and potassium ferricyanide (1 % w/v). The resulting mixture was incubated at 50 °C for 20 min followed by addition of 2.5 ml of trichloro acetic acid (10 % w/v) solution. The mixture was centrifuged at 3000 rpm for 10 min to collect the upper layer. 2.5 ml of solution from upper layer was mixed with 2.5 ml of distilled water and 0.5 ml of ferric chloride (0.1 % w/v) solution. The absorbance of the solution was then measured at 700 nm. Catechin was used for comparison.

### Determination of DPPH radical scavenging activity

DPPH radical scavenging activity of the different extracts of *V. roxburghii* was determined according to the method described by Choi *et al.* [38] with slight modifications. 2 ml of methanol solution of plant extract or reference standard at different concentration was mixed with 3 ml of methanol solution of DPPH (0.135 mM) into the test tube. The reaction mixture was incubated at room temperature for 30 min in dark place to complete the reaction. The absorbance of the solution was measured spectrophotometrically at 517 nm. DPPH free radical scavenging ability (%) was calculated by using the formula:

$$ \left[\left({\mathrm{A}}_{\mathrm{absorbance}\ \mathrm{of}\ \mathrm{control}}\hbox{--}\ {\mathrm{A}}_{\mathrm{absorbance}\ \mathrm{of}\ \mathrm{sample}}\right)\ /\ {\mathrm{A}}_{\mathrm{absorbance}\ \mathrm{of}\ \mathrm{control}}\right] \times 100 $$

### Determination of hydroxyl radical scavenging activity

The activity of the different *V. roxburghii* extracts in the scavenging of hydroxyl free radical was determined by the method as described by Elizabeth *et al.* [39] with a slight modification. In brief, plant extract or reference compound at different concentration was mixed with a reaction mixture contained, in a final volume of 1 ml: 2-deoxy-2-ribose (2.8 mM); KH_2_PO_4_-KOH buffer (20 mM, pH 7.4); FeCl_3_ (100 μM); EDTA (100 μM); H_2_O_2_ (1.0 mM); and ascorbic acid (100 μM). The mixture was then incubated for 1 h at 37 °C and 0.5 ml of the reaction mixture was was heated at 90 °C for 15 min after addition of 1 ml of 2.8 % TCA and 1 ml of 1 % aqueous TBA to develop the color. After cooling, the absorbance was measured at 532 nm against an appropriate blank solution. Hydroxyl radical scavenging ability (%) was calculated by using the formula:

$$ \left[\left({\mathrm{A}}_{\mathrm{absorbance}\ \mathrm{of}\ \mathrm{control}}\hbox{--}\ {\mathrm{A}}_{\mathrm{absorbance}\ \mathrm{of}\ \mathrm{sample}}\right)\ /\ {\mathrm{A}}_{\mathrm{absorbance}\ \mathrm{of}\ \mathrm{control}}\right] \times 100 $$

### Determination of lipid peroxidation inhibition activity

The ability of the different extracts of *V. roxburghii* to inhibit lipid peroxidation was determined according to the method as described by Liu *et al.* [40] with a slight modification. Rat brain was homogenized with a homogenizer in ice-cold phosphate buffer (50 mM, pH 7.4) with 0.15 M KCl to produce a 1/10 homogenate. The homogenate was centrifuged at 10,000 *g* for 15 min at 4 °C and the resulting supernatant was used as liposome for *in vitro* lipid peroxidation assay. 1 ml of 0.15 M KCl and 0.5 ml of brain homogenates and different concentrations of plant extract were mixed and the peroxidation reaction was initiated by adding 100 ml of 0.2 mM ferric chloride. After incubation at 37 °C for 30 min, the reaction was stopped by adding 2 ml of ice-cold HCl (0.25 N) containing 15 % trichloroacetic acid (TCA), 0.38 % TBA, and 0.5 % BHT. The reaction mixtures were heated at 80 °C for 60 min. The samples were cooled, centrifuged and the absorbance of the supernatants was measured at 532 nm. The percentage of inhibition of lipid peroxidation was calculated by the formula:

$$ \left[\left({\mathrm{A}}_{\mathrm{absorbance}\ \mathrm{of}\ \mathrm{control}}\hbox{--}\ {\mathrm{A}}_{\mathrm{absorbance}\ \mathrm{of}\ \mathrm{sample}}\right)\ /\ {\mathrm{A}}_{\mathrm{absorbance}\ \mathrm{of}\ \mathrm{control}}\right] \times 100 $$

### Determination of cholinesterase (ChE) inhibitory activities

The acetylcholinesterase (AChE) and butyrylcholinesterase (BChE) inhibitory assay were performed according to the colorimetric method of Ellman *et al.* [41]. For the AChE enzyme source, rat brain was homogenized in a homogenizer with 5 volumes of a homogenation buffer [10 mM Tris–HCl (pH 7.2), which contained 1 M NaCl, 50 mM MgCl_2_ and 1 % Triton X-100], and centrifuged at 10,000 *g* for 30 min. The resulting supernatant was used as an enzyme source. For the BChE enzyme source, human blood from anonymous healthy men subject (25 years) was provided by the Rajshahi Univresity Medical Center, Rajshahi, Bagladesh and collected in EDTA treated (1 mg/ml) glass tubes. The tubes were centrifuged at 2000 g for 10 min to eliminate the red blood cells. The resulting plasma (supernatant) was then recuparated, diluted (1/200) with 50 mM phosphate buffer (pH 7.4) and was used immediately for studying butyrylcholinesterase activity.

The rates of hydrolysis by acetylcholinesterase were monitored spectrophotometrically. Each extract or standard (500 μl) was mixed with an enzyme solution (200 μl) and incubated at 37 °C for 15 min. Absorbance at 405 nm was read immediately after adding an Ellman’s reaction mixture [3.5 ml; 0.5 mM acetylthiocholine, 1 mM 5, 5′-dithio-bis (2-nitro benzoic acid)] in a 50 mM sodium phosphate buffer (pH 8.0) to the above reaction mixture. Reading was repeated for 10 min at 2 min intervals to verify that the reaction occurred linearly. The blank reaction was measured by substituting saline for the enzyme. Donepezil was used as positive control. The percentage inhibition of acetylcholinesterase activity was calculated using the following formula:

$$ \left[\left({\mathrm{A}}_{\mathrm{absorbance}\ \mathrm{of}\ \mathrm{control}}\hbox{--}\ {\mathrm{A}}_{\mathrm{absorbance}\ \mathrm{of}\ \mathrm{sample}}\right)\ /\ {\mathrm{A}}_{\mathrm{absorbance}\ \mathrm{of}\ \mathrm{control}}\right] \times 100 $$

Assessment of BChE inhibition was performed as described above except that the enzyme solution was 50 μl and acetylthiocholine iodide (ACh) was replaced by butyrylthiocholine iodide (BCh). Galantamine was used as positive control. The percentage inhibition of butyrylcholinesterase activity was calculated using the same formula as mentioned above for acetylcholinesterase activity.

### Isolation of active compound from the chloroform extract

The chloroform extract (6.0 g) of *V. roxburghii* was fractionated by silica gel 60 (Merck, Germany) column chromatography using n-hexane, chloroform and methanol gradient system with gradually increasing polarity. Fractions of 25 ml were collected and concentrated. All eluted fractions were monitored by thin layer chromatography (TLC) and visualized under UV light (254 nm). Fractions with antioxidant activity caused by the same compounds (having the same R_f_ values) were combined. A total of five fractions (CLC1, CLC2 CLC3, CLC4 and CLC5) were obtained. The most active fraction CLC1 (1.1 g) was further separated by preparative thin layer chromatography using n-hexane-acetone (6:4) as the mobile phase to yield compound 1 (14 mg).

Spectral analysis for the compound 1 was achieved by using Jeol-Ex 400 MHz and FT-NMR spectrometers for ^1^H and ^13^ C-NMR spectra in CDCL_3_. The structure of the compound was confirmed by comparison of its spectral data with those published in the literature [42].

Gigantol (1): colorless gum, UV (in MeOH) λ_max,_ 226 and 282 nm. ^1^H-NMR (CDCl_3_): δ_H_ 6.81 (1H, d, *J* = 7.8 Hz), 6.68 (1H, d, *J =* 7.8 Hz), 6.62 (1H, s), 6.60 (1H, s), 6.24 (1H, s), 6.29 (1H, s), 2.82 (2H, m), 2.79 (2H, m), 3.82 (3H, s) and 3.74 (3H, s). ^13^C-NMR (CDCl_3_): δ_C_ 160.90 (C-5′), 156.67 (C-3′), 146.30 (C-5″), 144.55 (C-1′), 143.81 (C-4″), 133.64 (C-1″), 121.02 (C-2″), 114.32 (C-3″), 111.21 (C-6″), 108.07 (C-6′), 106.83 (C-4′), 99.06 (C-2′), 55.90 (C5″-OCH_3_),, 55.27 (C5′-OCH_3_), 38.29 (C-2), and 37.26 (C-1).

### Statistical analysis

All analyses were carried out in triplicates. Data were presented as mean *±* SD. Free R-software version 2.15.1 (http://www.r-project.org/) and Microsoft Excel 2007 (Roselle, IL, USA) were used for the statistical and graphical evaluations. Significant differences (*P*-value <0.05) between the means were determined using the *t*-test.

## Results and discussion

The practice of traditional medicine has described a wide range of plants and herbs which may be used in the treatment of AD. Such plants can play important roles in providing alternative medicine, adjuncts to existing therapies, and in the discovery of new pharmacological compounds. Indeed, one of the most widely used anti-AD drug, galantamine is derived from the plant [43]. *V. roxburghii*, an epiphyte has been used in Unani traditional medicine as tonic to brain and liver and also used by the local people of Bangladesh to treat nervous system disorders [24–26]. In this investigation, we evaluated the different extracts of *V. roxburghii* for cholinesterase inhibitory and antioxidant activities.

### Phytochemical analyses

The four different extracts of *V. roxburghii* viz. petroleum ether, chloroform, ethylacetate and aqueous extracts were analyzed qualitatively for the presence or absence of phytochemicals. The extracts showed the presence of tannins, phenolics and flavonoids, phytosterols, alkaloids and saponin (Table 1). Quantitative analyses (Table 2) revealed that the chloroform extract contained the highest amount of phenolics and flavonoids followed by ethylacetate, aqueous and petroleum ether extract.

**Table 1:** Qualitative phytochemical screening of different extracts from Vanda roxburghii

	PEF	CLF	EAF	AQF
Tannins	-	-	-	++
Flavonoids	+	+++	++	+
Phenolic compounds	+	+++	++	+
Alkaloids	-	-	-	+
Saponins	-	-	-	++
Phytosterols	++	+	-	-

Here, + = Present in mild amount, ++ = Present in moderate amount, +++ = Present in large amount, − = Absence

*PEF* petroleum ether fraction, *CLF* chloroform fraction, *EAF* ethylacetate fraction, *AQF* aqueous fraction

**Table 2:** Total phenolic and flavonoid contents and antioxidant activity of different extracts from Vanda roxburghii

Sample	TPC	TFC	DPPH	OH	FRAP
	(mg GAE/g dried extract)	(mg CE/g dried extract)	IC_50_ (μg/mL)	IC_50_ (μg/mL)	(absorbance at 100 μg/mL)
PEF	23.6 ± 0.21^(a)^	67.9 ± 1.35^(a)^	35.02 ± 1.56^(d)^	23.91 ± 1.58^(c)^	0.810 ± 0.02^(b)^
CLF	85.9 ± 1.03^(d)^	300.1 ± 0.61^(d)^	5.76 ± 0.42^(a)^	7.96 ± 0.61^(a)^	1.397 ± 0.04^(d)^
EAF	66.7 ± 0.73^(c)^	164.44 ± 0.85^(c)^	18.70 ± 0.78^(b)^	11.13 ± 0.72^(a)^	1.237 ± 0.04^(c)^
AQF	36.3 ± 0.60^(b)^	112.1 ± 0.95^(b)^	24.86 ± 1.12^(c)^	15.98 ± 0.86^(b)^	0.333 ± 0.01^(a)^
CA	-	-	4.55 ± 0.33^(a)^	9.45 ± 0.57^(a)^	1.48 ± 0.03^(e)^

Means in each column with different subscript letters (a b, c, d, e) differ significantly (*P* <0.05)

*PEF* petroleum ether fraction, *CLF* chloroform fraction, *EAF* ethylacetate fraction, *AQF* aqueous fraction, *CA* catechin, *TPC* Total phenolic content, *TFC* Total flavonoid content, *OH* Hydroxyl radical scavenging, *FRAP* Ferric reducing antioxidant power

### Cholinesterase inhibitory activity

**Fig. 1:**
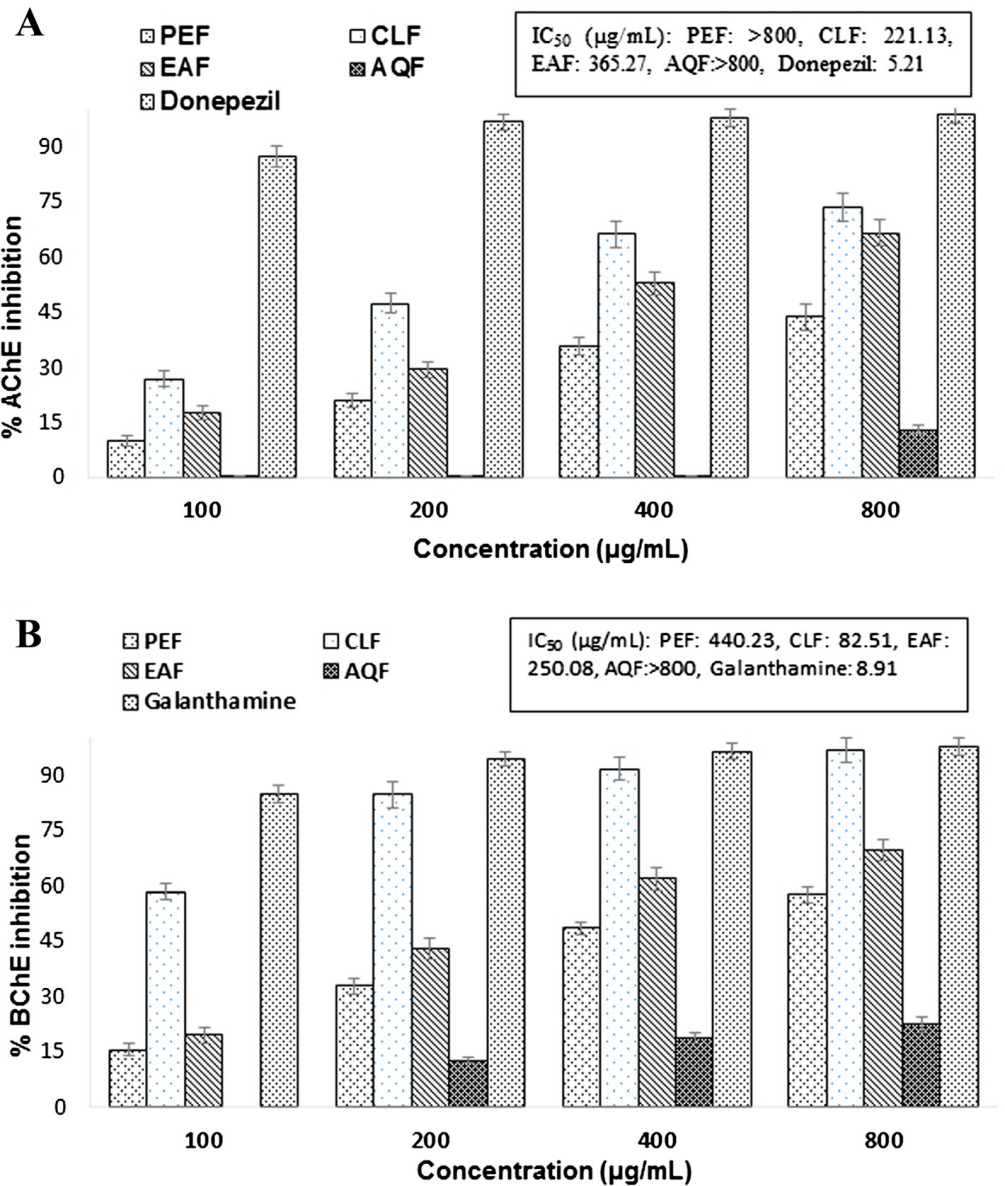
Percentage of inhibition of acetylcholinesterase (**a**) and butyrylcholinesterase (**b**) activity at different concentration of *V. roxburghii* extracts and the reference standard donepezil or galantamine. Standard was used for comparison. Results represent mean ± SD (n = 3, (P < 0.05). PEF, petroleum ether fraction; CLF, chloroform fraction; EAF, ethylacetate fraction; AQF, aqueous fraction

Because the cholinergic neurotransmitter acetylcholine is reduced in AD, inhibitor of cholinesterases has been shown to augment the activity of surviving cholinergic neurons in patients with AD, resulting in improvement of memory and cognition. All the cholinesterase inhibitors currently licensed for AD inhibit acetylcholinesterase and, to a varying extent, butyrylcholinesterase [11, 12]. The ability of *V. roxburghii* to inhibit both AChE and BChE, was evaluated by the widely accepted modified Ellman’s method [41] and the results have been shown in the Fig. 1. All the tested extracts except aqueous extract and petroleum ether extract inhibited the activity of AChE and BChE, respectively in a dose dependent manner. The results revealed that the chloroform extract displayed the highest inhibitory activity against both the cholinesterases, AChE and BChE. The IC_50_ values of the extract for AChE and BChE were found to be 221.13 μg/ml and 82.51 μg/ml, respectively, indicating that the chloroform extract has higher specificity for BChE. The activity of the chloroform extract was found to be strong when compared with the other extracts, but moderate when compared with that of the reference standard donepezil or galantamine, currently used as AD drugs. It has been reported earlier that dual inhibition of AChE and BChE might improve the signs of AD and symptoms owing to the key role of BChE in hydrolysis of acetylcholine [44]. However, our results demonstrated the dual cholinesterase inhibitory activity of *V. roxburghii*.

### Antioxidant activity

Antioxidant activity of plant extracts cannot be evaluated by a single method. Therefore, commonly accepted assays were used to assess the antioxidative effect of the different extracts of *V. roxburghii*. In a preliminary study, the antioxidant activity of the different extractives was evaluated employing ferric reducing antioxidant power (FRAP) and DPPH radical scavenging activity. Both assays demonstrated the potential antioxidant activity of the chloroform extract which was close to the activity of the reference standard catechin (Table 2). Chloroform extract showed the highest activity with an absorbance of 1.39 at 100 μg/ml concentration, which was close to the activity of the reference standard catechin that gave an absorbance of 1.48 at the same concentration. In DPPH radical scavenging, the IC_50_ of chloroform extract and catechin was found to be 5.76 and 4.55 μg/ml, respectively. We then extended our study employing the assays that are more relevant *in vivo*. Hydroxyl radicals are the major reactive oxygen species that severely damage the neurons in AD. Our results (Table 2) revealed that the chloroform extract has potential hydroxyl radical scavenging activity with an IC_50_ of 7.96 μg/ml, which appeared to be more potent than that of the reference standard catechin used in this study whose IC_50_ was found to be 9.45 μg/ml under the same condition.

**Fig. 2:**
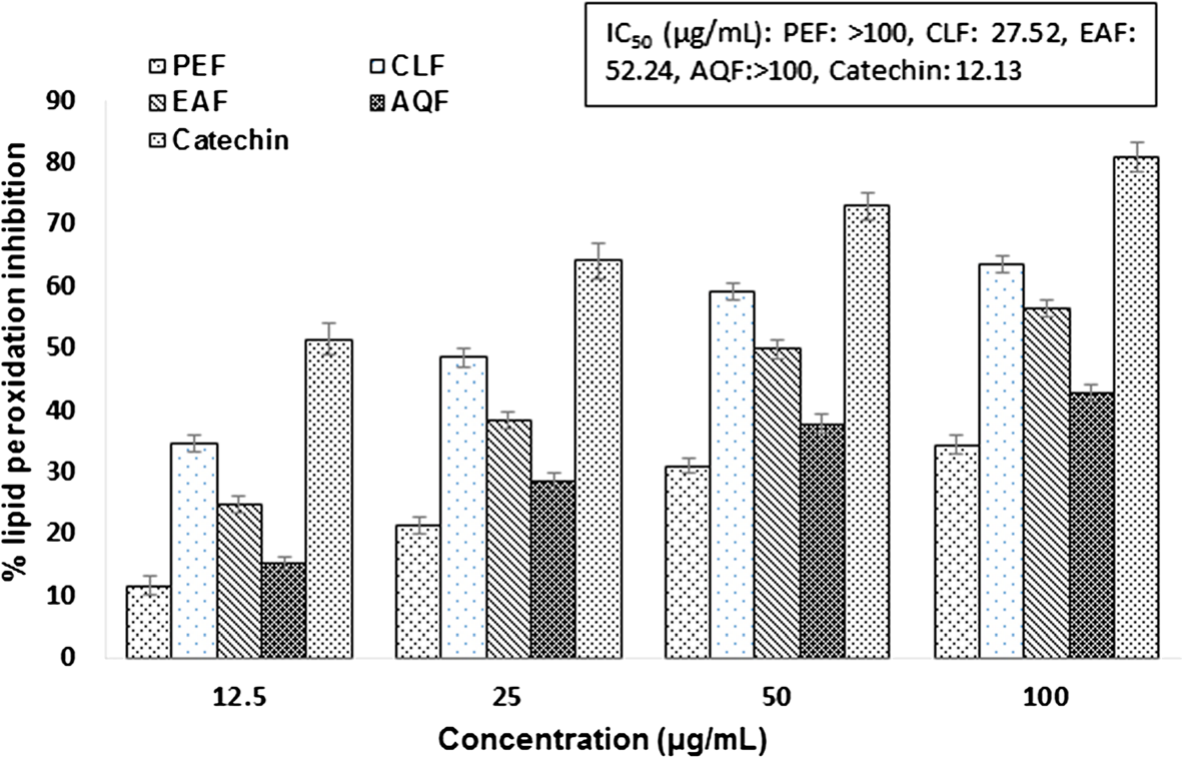
Percentage of lipid peroxidation inhibition at different concentration of *V. roxburghii* extracts and the reference standard catechin. Standard was used for comparison. Results represent mean ± SD (n = 3, (P < 0.05). PEF, petroleum ether fraction; CLF, chloroform fraction; EAF, ethylacetate fraction; AQF, aqueous fraction; CA, catechin

Oxidative damage in AD predominantly manifests as lipid peroxidation and increased lipid peroxidation has been observed in the brain of AD [14, 45, 46]. Lipid peroxidation products can be measured by using thiobarbituric acid [47]. Our results (Fig. 2) showed that the chloroform extract exhibited the highest activity among the extracts tested in the inhibition of brain lipid peroxidation with an IC_50_ value of 27.52 μg/ml. Taken together, our results demonstrated the potential antioxidant activity of the chloroform extract of *V. roxburghii.*

### Isolation of active compound from the chloroform extract of *V. roxburghii*

**Fig. 3:**
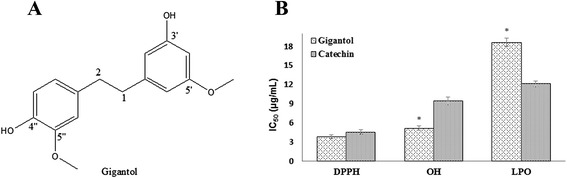
Chemical structure of gigantol (**a**) and its IC_50_ (mean ± SD) values for DPPH and hydroxyl radical (OH) scavenging and for inhibition of lipid peroxidation (LPO). * values significantly different (P < 0.05) from the reference standard catechin

**Fig. 4:**
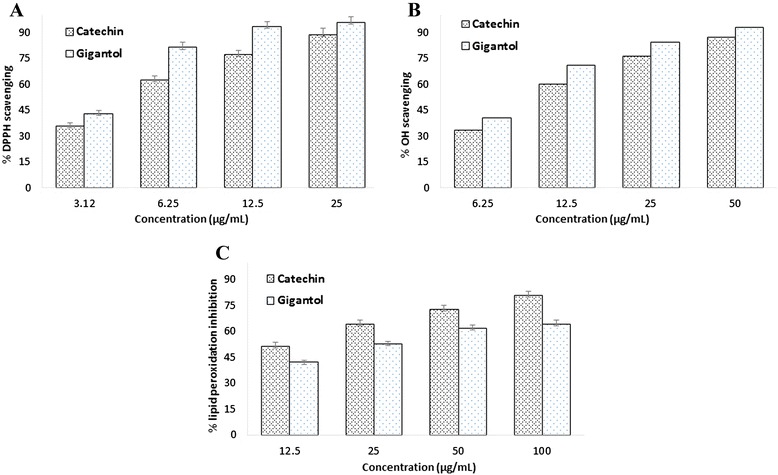
Percentage of DPPH (**a**) and hydroxyl (OH) radical (**b**) scavenging activity and lipid peroxidation inhibitory activity (**c**) at different concentration of gigantol and the reference standard catechin. Standard was used for comparison. Results represent mean ± SD (n = 3, (P < 0.05)

Since the chloroform extract was found to be the most active fraction, it was subjected to bioassay-guided separation using column chromatography and preparative thin layer chromatography that led to the isolation of a phenolic component from the active subfraction. The component was identified as gigantol by comparing its ^1^H NMR and ^13^C NMR spectra with the literature values [42]. Antioxidant activity of gigantol was again assayed in the same *in vitro* system. As shown in Figs. 3 and 4, gigantol was found to display strong radical scavenging activity which was consistent with the previous report [32] and exhibited inhibition of lipid peroxidation in rat brain homogenates. Both the radical scavenging and lipid peroxidation inhibitory activities of gigantol were shown to be dose-dependent. The compound did not show any activity against the cholinesterases.

Gigantol, a bibenzyle compound has been found to be an important constituent in the orchid plants and studied for a number of biological activities. Simmler et al. (2011) showed that gigantol from *V. cerule* displayed antioxidant and anti-inflammatory properties on HaCaT cell by neutralizing the intracellular ROS and by reducing the expression of COX-2, thereby reducing PGE-2 production [32]. In a previous report [48], gigantol from *Cymbidium goeringii* has been shown to possess potent inhibitory effects on LPS-induced nitric oxide (NO) and prostaglandin E2 (PGE-2) production in RAW 264.7 macrophage cells. The compound is also reported to reduce carrageenan-induced inflammation in rats [49]. Several lines of evidence proposed that inflammation may play a significant role in the pathogenesis of AD. It has been shown that the deposition of amyloid in AD brain brings about activation of micriglia and astrocytes, initiating a proinflammatory cascade that results in the release of potentially neurotoxic substances, cytokines, and other related compounds, bringing about degenerative changes in neurons [50]. Since gigantol has potential in scavenging free radicals, reducing lipid peroxidation (our study) and inflammation, suggesting that it may be a useful candidate drug in the treatment of AD and thus warrants testing in a animal model.

## Conclusions

Results of the present study clearly demonstrated that the chloroform extract of *V. roxburghii*, possibly due to its polyphenolic compounds, possess a combination of antioxidant properties and cholinesterase inhibitory activities which support its traditional utilization in Bangladesh in the treatment of AD. In a reference survey, no reports relating to the antioxidant and cholinesterase inhibitory activities of *V. roxburghii* has extracts have been found so far. Besides these, gigantol, a bibenzyl stilbenoid was isolated from the chloroform extract as the potent antioxidant principle.
